# Acute Appendicitis: Is Removal of a Normal Appendix Still Existing and can we Reduce its Rate?

**DOI:** 10.4103/1319-3767.61242

**Published:** 2010-04

**Authors:** Jyotindu Debnath

**Affiliations:** Department of Radiodiagnosis and Imaging, 167 Military Hospital, India. E-mail: jyotindu_debnath@rediffmail.com

Sir,

I read with great interest the article written by Dr. Gamal Khairy[1] titled ‘Acute Appendicitis: Is Removal of a Normal Appendix Still Existing and Can We Reduce Its Rate?’ published in the Jul-Sep 2009 issue of the Saudi Journal of Gastroenterology. The author highlights the importance of clinically based diagnosis of acute appendicitis. The author comments about the futility of routine CT scan in the diagnosis of acute appendicitis based on the reports of 3 (5.5%) out of 54 patients of negative appendectomy who had undergone preoperative CT scan. Is it justified to comment on the diagnostic value of CT scan based on reports of only 3 patients? Literature has abundant studies having proponents as well as opponents regarding utility of CT scan for appendicitis. I agree with the author that clinical judgment is of paramount importance in the diagnosis and management of acute appendicitis even today and liberal use of CT scan should be strongly discouraged. The pitfalls of CT scan need to be understood,[2] and the potential radiation burden to the patient need not be overemphasized. However, I would like to mention here that imaging has a definite and well-established role in the diagnosis and management of appendicitis. A clinician will always face the dilemma of balancing between early appendectomy (to prevent perforation) and negative appendectomy. Laboratory investigations, though useful, are nonspecific. Here comes the role of definitive imaging studies. It is surprising that the article does not even mention about ultrasonography being done in any of the patients. Despite relatively low sensitivity of ultrasonography, it provides a very high specificity, which possibly could have addressed the author's primary concern of how to reduce negative appendectomy rates. Ultrasonography can be carried out with minimum delay and can yield pertinent and surgically relevant information like confirmation of diagnosis of appendicitis, status of inflamed appendix, location and orientation of appendix, presence or absence of any associated complications, adhesions, free fluid, etc., besides suggesting alternative diagnoses, which helps in appropriate surgical planning. Today, what we need is a proper diagnostic algorithm and triage of patients of appendicitis with regard to the choice of investigations. Dedicated appendiceal ultrasonography by an experienced sonologist should be the first imaging modality in suspected cases of acute appendicitis.[3,4] In case the sonographic study is equivocal or negative and the clinical suspicion is very strong, then the patient may undergo selective CT scan as per protocol or may even be taken up for surgery at the discretion of the treating surgeon.
